# Odontological Society of Pennsylvania

**Published:** 1889-01

**Authors:** I. G. Baumgardner


### Reports of Society Meetings.

### ODONTOLOGICAL SOCIETY OF PENNSYLVANIA.

NOVEMBER, 1888.

ESPECIALLY REPORTED FOR "THE INTERNATIONAL," BY I. G. BAUMGARDNER, D.D.S.

Discussion on Dr. Truman's paper, entitled "Shock in Relation to Dental Operations."

*Dr. Chupein*—I quite agree with Dr. Truman in his views about the worn-out feeling which we frequently have after protracted operations, as I was suffering from this kind of "shock" when my day's work was ended. We often tax our strength in attending to more patients than we are able; yet, when people come to us suffering, and seek relief at our hands, we are prone to do this in the desire to do all we can for them.

*Dr. Sudduth*—There is one interesting phase of the question to which I have given considerable thought, and that is the peculiar effects of psychical influences. The mind certainly has a marked influence over the body in the performance of its functions. I remember, when studying in the hospital at Vienna, the case of a servant girl who came to the hospital complaining of a tired feeling, and who for four weeks proved an entire enigma as to the nature of her disease. Neither did the autopsy reveal anything. Our only clue was her history as recited by her friends, which was that two years previous she had been led astray, but had borne up, under promise of marriage, until within a few days previous to her admission to the hospital. Her decline and final collapse resulting in her death was attributed to the news coming to her notice of the marriage of her betrayer to another woman. Another case was that of a young lady who received word of the death of a near relative while at the dinner-table, when she had eaten heartily. Immediately the function of digestion was stopped, and the food remained in her stomach undigested for a long time, and it required a regular course of treatment to relieve the system of the undigested food. That was an instance of the mental action inhibiting the function of digestion. The third case occurred in my own family. I filled some small cavities for my little girl at my old office in Bloomington, Ill., this summer. They were quite sensitive, but she was brave, and while they were only filled temporarily, yet the strain was such as to cause an appreciable rise in temperature that evening, although the child was perfectly healthy. In practice, I never made an appointment for longer than one hour or an hour and a half. The change of patients is refreshing to the operator, and not so tiresome when confined to short engagements. There is a magnetic interchange from patient to operator that must be taken into account.

*Dr. Thomas*—I was greatly interested in Dr. Truman's paper, particularly so, because he recognizes the effect of shock in dental operations. Many dentists, formerly more than at present, seem to have no consideration, personally, for the patient, and I can endorse heartily Dr. Truman's suggestion that the time of sittings be shortened for patients of certain temperaments and nervous conditions. My specialty, as you all know, is extraction, and I have repeatedly had patients present themselves, who were amply able to pay for dental care, and who have spent large sums upon the preservation of their teeth, and yet their mouths were in a deplorable condition. They would come with the full determination to have their teeth extracted as they became troublesome, and declare that under no circumstance would they submit further to the strain and shock of dental manipulation. I know of several instances, but one in particular, of a young lady who, every time she went to the dentist for filling, would go home and remain in bed from three or four days to a week. I am happy to say that this difficulty has been largely removed since the more general introduction of plastics for fillings.

Dr. Truman speaks of the effects of shock in extraction, which we all know is a serious matter; and when not using an anæsthetic it is, no doubt, better to proceed cautiously, and do no more than the patient ought to bear; but when nitrous oxide is used, I do not agree that division of the operation is advantageous. I sometimes have patients sent to me with directions from their dentist or physician to extract only one or two teeth at one time, instructing them to return in a week or so to have a like number extracted, and so on until all that are condemned are removed. I do not think this is the best course. When a patient is under an anæsthetic, it does not matter whether you take one or a dozen out; and it has been my experience that a patient will endure the larger number all at once a great deal better than by dividing. The immediate effects show very little, if any, more symptoms of shock, and the relief of mind at having the operation completely finished adds a buoyancy to their nervous system which hastens recovery, while, on the other hand, they have the inconvenience of the sore places, and the constant dread of having to go through the ordeal again. This causes such depression that I have found it advisable more than once to defer extraction until conditions became more favorable.

This would tend to demonstrate that preceding nervous excitement and dread in anticipation of an operation are as depressing to some nervous conditions as the actual operation.

Mention is made of a case cited in the American System of Dentistry of a robust man declining rapidly in health after taking nitrous oxide. It is said that he lost forty pounds of flesh in a short space of time, and finally died of diabetes. I doubt very much that the gas caused the disease in his case. If it will do so in one, why not in another? Just think of the thousands upon thousands who have now taken nitrous oxide, and how extremely rare is a charge made that it has acted injuriously. I have in my own practice had a few complaints of supposed ill effects, but I have never met a case which was not readily and satisfactorily accounted for otherwise.

*Dr. C. N. Pierce*—I have had great pleasure in listening to Dr. Truman's paper. We cannot too strongly urge the necessity for short sittings in our operating chairs. One hour to one and a half is my limit, except in very exceptional cases. Protracted operations not only weary, but exhaust, the nervous force of both patient and operator. The essay tells the story plainly and emphatically, and its admonitions are valuable to our patients and ourselves.

*Dr. Faught*—The paper of Dr. Truman is certainly a valuable addition to our literature on the subject, and is one that concerns both our patients and ourselves. In discussing the subject I will divide my remarks into three heads. First: *Shock to those under operations.*—We are too prone, I think, simply to place patients in the chair without administering where particularly indicated, as is our right, medicinal agents both before and after the operation for the reduction of the nervous strain. I have been at fault in this myself, but for the past two years have given the matter careful study. We ignore the powers which lie in the course of proper medical treatment, and rely too much upon the supposition that the patient will recover physiologically. Second: *Shock to those under anæsthetics.*—Dr. Truman spoke of extracting six teeth at a time—that is all very right. In my opinion, we have no right to extract any teeth in Philadelphia without first recommending the patient to take the gas as administered by our skilled specialists. It is a grave question whether we should, as practitioners, do otherwise, knowing patients will suffer from it, when we have a remedy for them. In Philadelphia this branch is reduced to a science, and we do best for our patients when we recommend them to the services of a specialist. Third: *Shock in regard to ourselves.*—The symptoms of this are only too familiar to us. There is, however, one boon we too much neglect; that is sunshine. We enter our offices with lights before the sun is up, and leave it after it has set, thus losing the stimulating action upon the pores and general system. We take the shady side of the street, and avoid the sun whenever possible. Out-of-door exercise may be indulged in with great benefit; but best of all is sunshine, which we as dentists sadly neglect. It is our duty to sun ourselves more.

*Dr. Guilford*—I am pleased to find that Prof. Truman has brought this important subject before us, and that he has treated it in such a scientific and practical manner. It is a matter that largely concerns both our patients and ourselves. Fortunately we do not confront the condition, so far as our patients are concerned, nearly so frequently as we formerly did, and this is due to greater enlightenment on our part, together with improved methods of operating. Dr. Thomas thinks that it is largely due to the greater prevalence of plastic filling; but to my mind the change in the methods of tooth-restoration in general practice have had quite as much to do with it. Where the operator formerly spent from four to six hours in building up a tooth-crown with gold foil, he now makes the hollow crown in his laboratory, and simply mounts it in his office. Most of the work of restoration in its various forms has, of late years, been transferred from the office to the laboratory, with a corresponding physical gain to both patient and operator.

Dr. Thomas has also spoken of the use of nitrous oxide gas as a preventive of shock. There can be no question of the fact that an anæsthetic, especially a mild one like nitrous oxide, will generally prevent shock that would have occurred without it. Many years ago a girl came to me to have a tooth extracted without gas. The tooth being a lower one I placed her in an ordinary chair, so that I could stand back of and over her. Before I had grasped the tooth she was taken with an epileptic fit. After recovery, I placed her in my operating chair, administered the gas, and extracted the tooth without any unpleasant results.

The question of shock also has its subjective side from a dental standpoint. We ourselves are subject to it in a mild form, although we do not like to give it that name. Instead of working until we are wearied, and then ceasing, most of us are apt to continue until we are exhausted. Our collapsed condition at the close of the day shows that our system has suffered from shock, and, where kept up from day to day for a long time, cannot fail to injuriously affect us.

There is another form of "shock," to which the essayist has not alluded, although he must be familiar with it; for it seems to be growing more prevalent each year. Its symptoms are: Flushing of the face, protrusion of the eye-balls, and a singular elevation of the eye-lids. In addition, the tongue and vocal chords are affected, causing the afflicted person to utter strange sounds and speak in an excited manner. There are some peculiar and characteristic features about it. One is that it does not always immediately follow the operation that was the occasion of it. It may manifest itself at once, or it may follow only after a considerable time has elapsed. Another peculiarity about it is that the effects of the shock do not always manifest themselves in the individual for whom the work was performed, although they often appear in some other member of the same family. When a child has been the patient, the shock is usually noticeable in the father; and its occurrence corresponds exactly in point of time with the arrival of the bill.

*Dr. Bennett*—I am very much pleased with the paper. This is a subject that has received too little attention. In operating we always inflict some degree of pain or excite the fear of it, the one being about as bad as the other when patients are young or nervous. Either pain or fear or both may produce some degree of shock or exhaustion. It is just as important with children to allay their fears as to relieve pain. The best thing we can do for many of these at the first sitting is to help them to get rid of their fears.

It is trite, but may be well to mention that the best means at all times to avoid shock and undue dread of the dental chair is to begin with the simple and least sensitive operations. We all know the good effects of first cleaning the teeth, especially with new patients. As for the others who may be fearful, or not capable of much endurance, we must, of course, depend on short sittings and a judicious use of remedies for sensitiveness in any of the tissues invaded by our operations.

(*To be continued.*)

